# Ruxolitinib in refractory acute and chronic graft-versus-host disease: a multicenter survey study

**DOI:** 10.1038/s41409-019-0731-x

**Published:** 2019-11-07

**Authors:** Virginia Escamilla Gómez, Valentín García-Gutiérrez, Lucía López Corral, Irene García Cadenas, Ariadna Pérez Martínez, Francisco J. Márquez Malaver, Teresa Caballero-Velázquez, Pedro A. González Sierra, María C. Viguria Alegría, Ingrid M. Parra Salinas, Cristina Calderón Cabrera, Marta González Vicent, Nancy Rodríguez Torres, Rocío Parody Porras, Christelle Ferra Coll, Guillermo Orti, David Valcárcel Ferreiras, Rafael De la Cámara LLanzá, Paula Molés, Kyra Velázquez-Kennedy, María João Mende, Dolores Caballero Barrigón, Estefanía Pérez, Rodrigo Martino Bofarull, Silvanna Saavedra Gerosa, Jorge Sierra, Marc Poch, María T. Zudaire Ripa, Miguel A. Díaz Pérez, Blanca Molina Angulo, Isabel Sánchez Ortega, Jaime Sanz Caballer, Juan Montoro Gómez, Ildefonso Espigado Tocino, José A Pérez-Simón, On behalf of the Grupo Español de Trasplante Hematopoyético (GETH)

**Affiliations:** 1 Department of Hematology of the University Hospital Virgen del Rocío, Instituto de Biomedicina (IBIS/CSIC/CIBERONC, CB16/12/00480), Universidad de Sevilla, Seville, Spain; 2 Department of Hematology, University Hospital Ramon y Cajal, IRYCIS, Madrid, Spain; 3 Complejo Asistencial Universitario de Salamanca-IBSAL, Centro de Investigación del Cáncer-IBMCC, Salamanca, Spain; 4 Hospital Santa Creu I Sant Pau, Barcelona, Spain; 5 Clinic University Hospital of Valencia, Valencia, Spain; 6 University Hospital, Granada, Spain; 7 Hospital of Navarra, Navarra, Spain; 8 University Hospital Miguel Servet, Zaragoza, Spain; 9 Hospital Niño Jesús, Madrid, Spain; 10 Instituto Catalá de Oncología Hospitalet, Barcelona, Spain; 11 Instituto Catalá de Oncología Germans Trias, Badalona, Spain; 12 University Hospital Vall d´Hebron, Barcelona, Spain; 13 Hospital La Princesa, Madrid, Spain; 14 Hospital La Fe, Valencia, Spain

**Keywords:** Drug development, Molecularly targeted therapy

## Abstract

Graft-versus-host disease is the main cause of morbidity and mortality after allogeneic hematopoietic stem cell transplantation. First-line treatment is based on the use of high doses of corticosteroids. Unfortunately, second-line treatment for both acute and chronic graft-versus-host disease, remains a challenge. Ruxolitinib has been shown as an effective and safe treatment option for these patients. Seventy-nine patients received ruxolitinib and were evaluated in this retrospective and multicenter study. Twenty-three patients received ruxolitinib for refractory acute graft-versus-host disease after a median of 3 (range 1–5) previous lines of therapy. Overall response rate was 69.5% (16/23) which was obtained after a median of 2 weeks of treatment, and 21.7% (5/23) reached complete remission. Fifty-six patients were evaluated for refractory chronic graft-versus-host disease. The median number of previous lines of therapy was 3 (range 1–10). Overall response rate was 57.1% (32/56) with 3.5% (2/56) obtaining complete remission after a median of 4 weeks. Tapering of corticosteroids was possible in both acute (17/23, 73%) and chronic graft-versus-host disease (32/56, 57.1%) groups. Overall survival was 47% (CI: 23–67%) at 6 months for patients with aGVHD (62 vs 28% in responders vs non-responders) and 81% (CI: 63–89%) at 1 year for patients with cGVHD (83 vs 76% in responders vs non-responders). Ruxolitinib in the real life setting is an effective and safe treatment option for GVHD, with an ORR of 69.5% and 57.1% for refractory acute and chronic graft-versus-host disease, respectively, in heavily pretreated patients.

## Introduction

Graft-versus-host disease (GVHD) is the main cause of morbidity and mortality after allogeneic hematopoietic stem cell transplantation (HSCT). Despite the use of standard prophylaxis 35–50% and 35–70% of HSCT recipients will develop acute (aGVHD) [1] and chronic GVHD (cGVHD) [2], respectively.

First-line systemic treatment consists of high doses of corticosteroids. Unfortunately, more than 50% of the patients will not respond adequately, thus requiring second-line treatment [3]. This subgroup of patients has an especially poor prognosis, with a significantly higher risk of treatment-related mortality [4]. Until recently, there were no approved therapies for GVHD treatment [5].

Ruxolitinib is an orally administered selective Janus Kinase (JAK) inhibitor approved for the treatment of myelofibrosis and polycythemia vera [6–9]. JAK inhibitors relieve symptoms related to an excess of proinflammatory cytokines in these patients [10, 11]. Due to the key role of JAK-STAT pathways on T cells activation, JAK inhibitors may reduce GVHD by inhibiting donor T-cell expansion and inflammatory cytokine production, regulatory T-cell (Treg) function and viability. Based on this background, Spoerl et al. [12] and Zeiser et al. [13] have reported the effectiveness of ruxolitinib to control GVHD in both mice and humans.

Several approaches have been evaluated as recue therapy within the second-line treatment. The difficulty in grading the severity (consequence of the high heterogeneity of the manifestations) and the treatment responses, as well as the sequential or concomitant treatment with several immunosuppressive drugs, makes it difficult to evaluate the effectiveness of any approach. In this context, the German group [13] has published data from a retrospective study in which 95 patients with moderate-severe GVHD refractory to steroids were treated with ruxolitinib. The overall response rates (ORR) were 44/54 (81%) and 35/41 (85%) for aGVHD and cGVHD respectively, with rates of up to 46% of complete responses (CR) in aGVHD. To assess long-term follow-up results, they collected data in a second analysis [14] from the same patients. Ongoing ORR was 22/54 (41%) and 10/41 (24%) after a median follow-up of 19 and 24 months for aGVHD and cGVHD groups. The 1-year overall survival (OS) was 62.4% (CI: 49.4–75.4%) and 92.7% (CI: 84.7–100%), respectively. Other authors, such as Khoury et al. [15], reported the outcomes of 19 patients with cGVHD who received salvage therapy with ruxolitinib. They described early partial responses (PR) in 18 out of 19 patients as well as a sustained steroid-sparing effect in 17 out of 19 patients.

On May 24, 2019, the Food and Drug Administration approved ruxolitinib (JAKAFI, Incyte Corporation) for steroid-refractory aGVHD in adult and pediatric patients 12 years and older [5]. Approval was based on Study INCB 18424-271 (NCT02953678), an open-label, single-arm, multicenter study of ruxolitinib that enrolled 49 patients with steroid-refractory aGVHD grades 2–4 (Mount Sinai Acute GVHD International Consortium criteria). Ruxolitinib was administered at 5 mg twice daily, and the dose could be increased to 10 mg twice daily. The trial’s primary endpoints were day-28 ORR. The median response duration was 16 days (95% CI: 9, 83), and the median time from day-28 response to either death or need for new therapy for aGVHD was 173 days (95% CI 66, NE).

In addition, Novartis Inc is running two large phase III trials of ruxolitinib vs best standard of care in steroid-refractory aGVHD and cGVHD. They are open-label studies in period of recruiting. However clinical data and outcomes are not available yet.

With this background, we analyzed the use of ruxolitinib in the treatment of GVHD within the Spanish Group of Hematopoietic Transplant and Cell Therapy (GETH) centers. Our data add evidence to the information available so far, on this new therapeutic strategy.

## Methods

### Study population

Between October 2015 to July 2017, 79 patients who underwent an HSCT and developed GVHD resistant to steroids received ruxolitinib. They were evaluated in this retrospective, observational, and multicenter study using data collected from 13 Spanish centers, including seven pediatric patients (<14 years). Off-label treatment with ruxolitinib and data analysis were approved by the Clinical Research Ethics Committee of the Hospital Universitario Ramón y Cajal, Spain.

The median age was 51 years (range, 0–73). The most frequent underlying diseases were: acute myeloid leukemia (38%), non-Hodgkin lymphoma (16.5%) and acute lymphoblastic leukemia (15.2%). The majority of patients received reduced-intensity conditioning regimens (57%). Patient baseline characteristics of the entire population are shown in Table 1. Of note, 53% and 55% of patients with aGVHD or cGVHD, respectively, have received three or more lines or prior therapy.

**Table 1:** Patient characteristics

	*N* (%)
Gender	
Male	48 (60.8)
Female	31 (39.2)
Age	
Median (range)	51 (0–73)
Underlying disease	
Acute myeloblastic leukemia	30 (38)
Acute lymphoblastic leukemia	12 (15.2)
Myelodysplastic syndrome	11 (13.9)
Multiple myeloma	3 (3.8)
Hodgkin disease	2 (2.5)
Non-Hodgkin disease	13 (16.5)
Myelofibrosis	4 (5.1)
Others	4 (5.1)
Disease status previous HSCT	
CR	47 (59.5)
PR	13 (16.5)
SD	11 (13.9)
Others	8 (10,1)
Type of transplant	
Related HLA identical donor	33 (41.7)
Haploidentical	7 (8.8)
Unrelated donor	39 (49.3)
Conditioning regimen	
Myeloablative	34 (43)
Reduced-intensity	45 (57)
Source	
Peripheral blood	75 (95)
Bone marrow	2 (2.5)
Umbilical cord	2 (2.5)

Acute GVHD (*n* = 23)	*N* (%)
Grades	
2–4	23 (100)
3–4	20 (87)
Organs involved	
Skin	16 (69.6)
Gut	21 (95.5)
Liver	13 (59.1)
Previous lines of therapy	
1	4 (18.2)
2	7 (31.8)
3	6 (26.1)
>3	6 (27.2)

Chronic GVHD (*n* = 56)	*N* (%)
NIH score	
Mild	0 (0)
Moderate	28 (50)
Severe	28 (50)
Organs involved	
Skin	44 (78.5)
Sclerotic changes	25 (41.1)
Resembling lichen planus	2 (3.6)
Rash	13 (23.2)
Hyper/hypopigmentation	3 (5.4)
Ichthyosis	3 (5.4)
Oral	33 (58.9)
Ocular	23 (41)
Gut	16 (28.6)
Liver	10 (17.8)
Lung	26 (45)
Joint mobility disfunction	22 (39.4)
Urinary tract	7 (12.5)
Esophageal membrane	2 (3.6)
Pericardial/pleural effusion	2 (3.6)
Nephrotic syndrome	1 (1.8)
Neuropathy	1 (1.8)
Microangiopathy	1 (1.8)
Polymyositis	3 (5.3)
Previous lines of therapy	
1	4 (7.1)
2	20 (35.7)
3	11 (19.6)
>3	21 (37.6)

The study was carried out in accordance with the principles of Declaration of Helsinki and received approval by an independent Clinical Research Ethics Committee. Written informed consent for collection data was obtained and signed from each patient after being treated with ruxolitinib. Confidentiality of data collection was preserved following local regulations (Organic Law 15/1999 of December 13, Protection of Personal Data [LOPD]). Likewise, Law 14/2007 on Biomedical Research was respected.

### Inclusion criteria and treatment plan

Patients undergoing HSCT in GETH centers with steroid-refractory GVHD treated with ruxolitinib were included in the analysis. Refractoriness of aGVHD was defined as “progression within 3–5 days of starting treatment or an incomplete response by 7–14 days. Refractory cGVHD was defined as “cGVHD of sustained severity during the last full month during which the patients had received the equivalent of prednisone 0.5 mg/kg or more per day or 1 mg/kg or more every other day”.

The severity of the disease was evaluated according to the International Bone Marrow Transplant Registry criteria for aGVHD [16] and according to the international consensus of National Institutes of Health (NIH) for cGVHD [17]. Patients were scored for their best response at any time after starting ruxolitinib. Treatment responses were considered when patients achieved CR or PR. Other types of responses were considered treatment failure.

Regarding aGVHD, CR was defined as the absence of symptoms related to the GVHD. The PR as improvement of at least one category of the severity of aGVHD in one organ without deterioration in any other. Treatment failure was defined as the lack of improvement of GVHD, deterioration in any organ, appearance of new symptomatology associated with GVHD or the need to start a new treatment for the control of the disease.

Regarding cGVHD, response assessment was performed following NIH criteria [18]. CR was defined as resolution of all manifestations related to cGVHD in a specific organ; PR as improvement in score from baseline reflecting genuine clinical benefit; and treatment failure as criteria for progression defined in NIH consensus. Discontinuation of ruxolitinib due to toxicity was not considered treatment failure. Histologic GVHD grading was performed on the basis of histopathology according to a published staging system for histology and clinical grading according to criteria for aGVHD or cGVHD [19].

### Study design

This is a retrospective, observational and multicenter study. Safety and efficacy data were analyzed in patients who have already been treated with ruxolitinib in the clinical practice under a compassionate use. The study did not imply a change in the therapeutic action or additional tests. The information source was patient´s clinical history in all cases. The study was performed within the hospital setting, with the participation of Departments of Hematology belonging to the Spanish Group of Hematopoietic Transplant and Cell Therapy (GETH) distributed throughout the national territory. Data were collected in a specific Electronic Case Report Form especially designed for the study.

### Statistics

Results were analyzed using the Statistical Package for the Social Sciences (SPSS PASW18). A *p* < 0.05 was considered statistically significant. OS was calculated in our study at one year with Stata/IC 15.0 program. Given that the objective of the study was merely descriptive, and therefore, there was not hypothesis to be confirmed, the sample estimation prior to the study was not necessary.

## Results

### Ruxolitinib in aGVHD

**Fig. 1:**
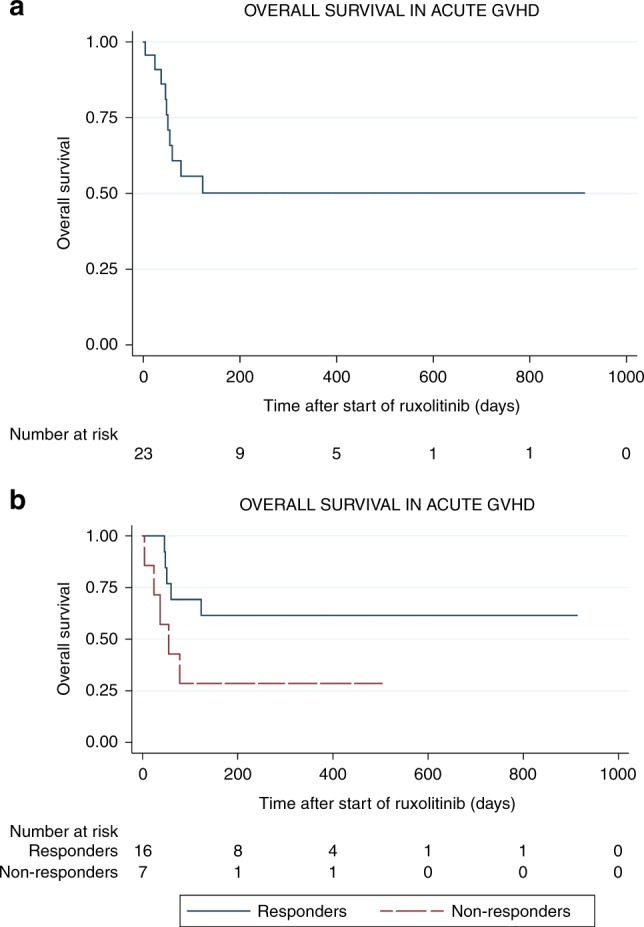
**a** Overall survival among patients with acute GVHD. **b** Overall survival in responders vs non-responder acute GVHD patients

Twenty-three patients received ruxolitinib for refractory aGVHD. All patients had grades 2–4 aGVHD and 20 patients (87%) had grades 3–4; the median number of previous lines of therapy was 3 (range 1–5). ORR was 69.5% (16/23) which was obtained after a median of 2 weeks of treatment (range: 0.5–4 weeks), and 21.7% (5/23) reached CR. Median follow-up was 78 days (range: 4–913). The median dose of ruxolitinib was 20 mg/day divided in two doses. Remarkably, we found no differences in treatment responses depending on the organs involved (Table 2). More specifically, 66.7% of patients with gastrointestinal GVHD did respond, 19% obtaining CR. The use of ruxolitinib allowed to taper steroids doses in 17/23 of patients (73.7%). Globally, overall survival at 6 months was 47% (CI: 23–67%) (Fig. 1a). Overall survival (OS) at 6 months in responders vs non-responders was 62% vs 28%, respectively (Fig. 1b).

**Table 2:** Ruxolitinib responses

Acute GVHD (*n* = 23)	ORR	CRR
Overall response	16/23 (69.5)	5/23 (21.7)
Response rate in grades 3–4	14/20 (70)	5/20 (25)
RR by organs		
Skin	11/16 (68.8)	3/16 (18.7)
Gut	14/21 (66.7)	4/21 (19)
Liver	9/13 (69.2)	3/13 (23)
RR ≥ 3 lines of treatment	9/12 (75)	
And aGVHV grades 3–4	8/11 (72.7)	2/11 (18.2)
And skin involvement	8/10 (80)	2/9 (20)
And gut involvement	8/11 (72.7)	1/11 (9)
And liver involvement	4/6 (66.7)	1/6 (16)

Chronic GVHD (*n* = 56)	ORR	CRR
Overall response	32/56 (57.1)	2/56 (3.5)
RR by grades		
Moderate	17/28 (60.7)	1/28 (3.5)
Severe	15/28 (53.5)	1/28 (3.5)
RR by organs		
Skin with sclerotic changes	14/25 (56)	0/25 (0)
Lung	16/26 (61.5)	2/26 (7)
Gut	9/16 (56.3)	2/16 (12)
RR ≥3 lines of treatment	17/32 (53.1)	2/32 (6.3)
And moderate plus severe	17/32 (53.1)	2/32 (6.3)
cGVHD		
And skin involvement with sclerotic changes	8/15 (53.3)	0/15 (0)
And lung involvement	10/14 (71.4)	2/14 (14.2)
And gut involvement	7/10 (70)	2/10 (20)

### Ruxolitinib in cGVHD

**Fig. 2:**
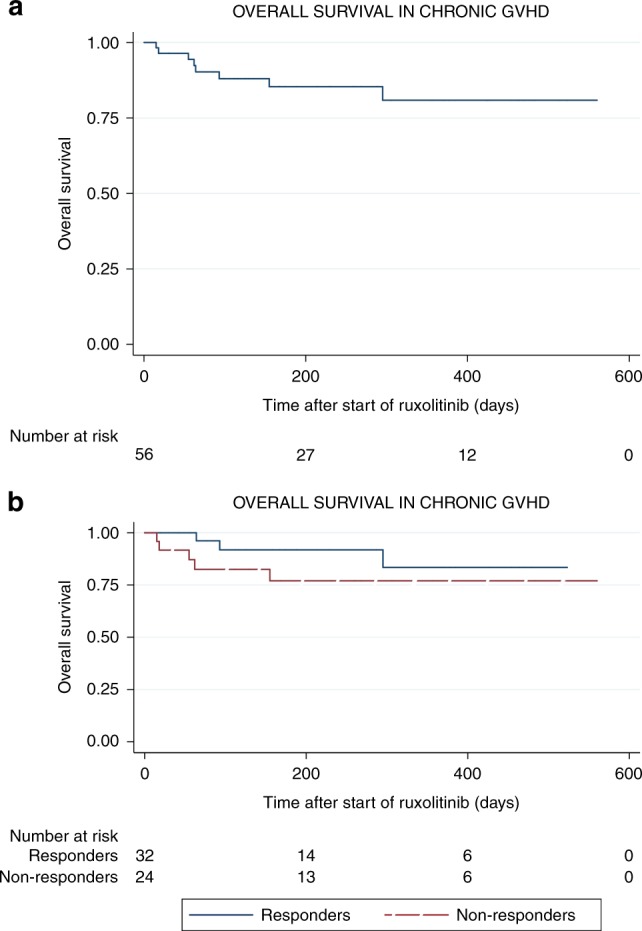
**a** Overall survival among patients with chronic GVHD. **b** Overall survival in responders vs non-responder chronic GVHD patients

Fifty-six patients were evaluated for refractory cGVHD. All patients had moderate (28/57, 50%) to severe (28/57, 50%) cGVHD. The median number of previous lines of therapy was 3 (range 1–10). ORR was 57.1% (32/56) with 3.5% (2/56) obtaining CR which was obtained at a median of 4 weeks of treatment (range: 1–24 weeks). Median follow-up was 181 days (range: 15–560). The median dose administrated was 20 mg daily divided in two doses. Again, no differences were found upon analyzing response rate by organs involved. Remarkably, ORR in patients with sclerotic changes was 56%, for those with lung involvement 61.5% and for those with gut involvement 56.3%. Responses for lung involvement were evaluated according to NIH scoring/staging/response assessment as part of standard clinical practice. Thirty-two patients (59.2%) could taper the doses of steroids. OS at 1 year was 81% (IC: 63–89) (Fig. 2a). OS at 1 year in responders vs non-responders was 83% vs 76%, respectively (Fig. 2b).

### Toxicities, relapse, and mortality

Cytomegalovirus (CMV) reactivation was observed both in aGVHD and chronic subgroups of patients while on treatment with ruxolitinib. Regarding aGVHD, CMV reactivation occurred in 12/23 (52.2%) patients, while in the cGVHD subgroup, it was observed in 11/56 (19.6%) patients. Nevertheless, when we analyzed CMV reactivation before ruxolitinib treatment was started, the incidence was similar or even higher: among patients with aGVHD: 12/23 (52.2%); and among patients with cGHVD: 15/56 (26%), indicating that ruxolitinib may not exert a significant increase in the risk of CMV reactivation. Monitoring by plasma CMV PCR was performed in all recipients and CMV reactivations were treated according to clinical practice. Since these data were retrospectively collected in different centers, there was not a uniform algorithm. Globally, it was defined as 2 confirmed PCR CMV tested above 600 copies. Those patients with confirmed reactivation received valgancyclovir (foscarnet in case of severy neutropenia) according to current recommendations.

Overall, 26 patients (32.9%) interrupted ruxolitinib due to: lack of response (14), cytopenias (three patients had thrombocytopenia, three anemia, three had both); infections (1); and other causes (2).

Regarding drug-related toxicities, only three patients discontinued ruxolitinib (Table 3). Causes for discontinuation in these patients were fungal infection, thrombocytopenia, and hepatic impairment. For 16 patients, it was sufficient with temporary suspension or dose reduction.

**Table 3:** Toxicities, adverse events, and malignancy relapse

Toxicities and adverse events	*N* = 26/79 (32.9%)
Infections	4 (5)
Fungal infection	2
Bacterial/viral infection	2
Cytopenia	14 (17.7)
Anemia	3
Leukopenia	2
Thrombocytopenia	5
Combinations	4
Others	8 (10.1)
Renal impairment	3
Hepatic impairment	3
Hypertension	1
Edema	1
Action	
Dose reduction	14 (17.7)
Temporary suspension	2 (2.5)
Discontinuation	3 (3.7)
No actions/Others	7 (8.8)
Malignancy relapse	1 (1.2)

Relapse of the underlying malignancy was only observed in one non ruxolitinib-responsive patient.

Globally, 18 patients (22.8%) died: 10/23 patients (43.5%) within the aGVHD and 8/56 patients (14.3%) within the cGVHD subgroup. Causes of death were: infections (10), refractory GVHD (6) and other causes (2).

We also analyzed bilirubin, alkaline phosphatase, creatinine and LHD levels before ruxolitinib was started. However, we did not find any biomarkers that could predict treatment responses. Median bilirubin, alkaline phosphatase, creatinine, and LHD levels were 1.5 mg, 117 U/L, 1 mg/dl, and 289 U/L respectively.

## Discussion

The development of novel approaches for the treatment of relapsed or refractory GVHD is an unmet medical need. In the current study, ORR among patients with aGVHD was 69.5% (16/23) with 21.7% (5/23) patients obtaining CR. Among patients with refractory cGVHD, ORR was 57.1% (32/56) with 3.5% (2/56) obtaining CR. It is worth mentioning that, in the current study, the response rate reached 56% for cGVHD with skin involvement and sclerotic features, which is promising considering the limited therapeutic options for these patients [20]. Similarly, lung involvement is one of the most severe features of cGVHD. We observed a response rate of 61% among these patients. Other studies using extracorporeal photopheresis have observed a response rate in the range of 33–63% [21]. In addition, considering that ruxolitinib is administrated orally, there might be some concerns regarding its absorption and biodistribution. Noteworthy, ORR reached 67 and 56% among patients with gastrointestinal aGVHD and cGVHD, respectively. Considering the retrospective nature of the study, we did not set a specific time point post ruxolitinib for GVHD assessment, but we based our analysis on the time of best response. The lack of a standardized time point to assess ORR is a limitation and could be considered a flaw in the methodology. Nevertheless, taking into account the median time to best response for aGVHD by day 14 and considering that late responses occurred up to day 27, our data does well represent the ORR occurring by day +28, which is currently considered the gold standard regarding the timing for aGVHD evaluation.

The German group [13] has reported data from a retrospective study in which 95 patients with moderate-severe GVHD refractory to steroids were treated with ruxolitinib. The ORR were 81% (44/54) and 85% (35/41) for aGVHD and cGVHD respectively, with rates of up to 46% (25/54) of CR in aGVHD and 7.3% (3/41) in cGVHD. OS rates at 6 months were 79% and 97%, respectively. Tapering of corticosteroids was possible in both aGVHD (17/23, 73%) and cGVHD (32/56, 57.1%) groups. In the current study, the median number of prior lines of treatment was 3 (1–5) among patients with aGVHD, and 3 (1–10) for patients with cGVHD. Accordingly, the response rates previously described were obtained in heavily pretreated patients, both in the current study as well as in the study by Zeiser et al., although ORR and CR rates were higher in the German study. Ongoing prospective randomized trials are required to confirm these data, although in both studies, the response rate is remarkable as compared with other approaches [22–32] and, furthermore, the toxicity profile was manageable in this fragile population.

The study led by Khoury et al. [15], reported outcomes of 19 patients with steroid-resistant cGVHD who received salvage ruxolitnib therapy. In their analysis they described early PR in 18 out of 19 patients. Of importance, they remark the reduction to physiologic doses or discontinuation of prednisone in ~90% of patients.

Recently, Incyte Corporation has announced positive results from its ongoing pivotal Phase 2 REACH1 trial for aGVHD. The study showed an ORR of 55% (*n* = 39/71) at day 28, and the best ORR at any time was 73% (*n* = 52/71), thus corroborating our findings. The most common treatment adverse events described were anemia (61%), thrombocytopenia (61%), and neutropenia (56%).

In the current study, the safety profile was satisfactory, with the most frequent side effects consisting of cytopenias and CMV reactivation. According to our data, CMV reactivation was observed in both aGVHD (52.2%) and chronic (19.6%) GVHD during the treatment. However, the analysis of CMV reactivation before starting ruxolitinib was even higher, suggesting that treatment with ruxolitinib might not increase the risk of CMV reactivation as suggested in other studies. Therefore, CMV copy numbers should be monitored as a standard procedure according to current guidelines for clinical practice in this heavily pretreated group of patients in order to administer preemptive treatment if required, not just because of an increased risk of reactivation related to the drug but because of patients characteristics.

Concerning other toxicities related to the treatment, we found cytopenias as the most frequent event. It is known that JAK–STAT pathways are essential for cytokine-mediated hematopoiesis [12]; that is the reason why thrombocytopenia and anemia are one of the major adverse effects of ruxolitinib that have been observed in other studies in myelofibrosis. In our study, only three patients discontinued ruxolitinib due to drug-related toxicities, indicating that the drug shows an excellent toxicity profile.

It is also worth mentioning that a higher immuno-suppression might lead to a potential increased risk of relapse of the underlying malignancy [33]. In our study, we did not observe any relapse among ruxolitinib-responsive patients. The only relapse observed in our series was seen in a patient who did not respond to ruxolitinib. Overall, the frequency of relapse was very low (1.2%) in comparison with other studies using other immunosuppressive drugs.

In summary, ruxolitinib in the real life setting has been shown as an effective and safe treatment option for GVHD patients, with an ORR of 69.5% and 57.1% for refractory aGVHD and cGVHD, respectively, among heavily pretreated patients. It is therefore a reasonable alternative to consider for the treatment of steroid-refractory aGVHD and cGVHD. Its effectiveness has been shown both in the improvement of GVHD as well as in the probability to spare the doses of steroids.

## References

[1] Jacobsohn DA, Vogelsang GB (2007). Acute graft versus host disease. Orphanet J Rare Dis.

[2] Socié G, Ritz J (2014). Current issues in chronic graft-versus-host disease. Blood.

[3] Martin P, Rizzo JD, Wingard JR, Ballen K, Curtin PT, Cutler C (2012). First- and second- line systemic treatment of acute graft-versus-host disease: recommendations of the American Society of Blood and Marrow Transplantation. Biol Blood Marrow Transplant.

[4] Westin JR, Saliba RM, De Lima M, Alousi A, Hosing C, Qazilbash MH (2011). Steroid-refractory acute GVHD: predictors and outcomes. Adv Hematol.

[5] Jaglowski SM, Blazar BR (2018). How ibrutinib, a B-cell malignancy drug, became an FDA-approved second-line therapy for steroid-resistant chronic GVHD. Blood Adv.

[6] Harrison C, Kiladjian JJ, Al-Ali HK, Gisslinger H, Waltzman R, Stalbovskaya V (2012). JAK inhibition with ruxolitinib versus best available therapy for myelofibrosis. NEnglJMed.

[7] Greenfield G, McPherson S, Mills K, McMullin MF (2018). The ruxolitinib effect: understanding how molecular pathogenesis and epigenetic dysregulation impact therapeutic efficacy in myeloproliferative neoplasms. J Transl Med.

[8] Harrison CN, Mead AJ, Panchal A, Fox S, Yap C, Gbandi E (2017). Ruxolitinib vs best available therapy for ET intolerant or resistant to hydroxycarbamide. Blood.

[9] Verstovsek S, Mesa RA, Gotlib J, Levy RS, Gupta V, DiPersio JF (2012). A double-blind, placebo-controlled trial of ruxolitinib for myelofibrosis. N Engl J Med.

[10] Takanori T (2014). JAK inhibitors: a home run for GVHD patients?. Blood.

[11] Choi J, Cooper ML, Alahmari B, Ritchey J, Collins L, Holt M (2014). Pharmacologic blockade of JAK1/JAK2 reduces GvHD and preserves the graft-versus-leukemia effect. PLoS One.

[12] Spoerl S, Mathew NR, Bscheider M, Schmitt-Graeff A, Chen S, Mueller T (2014). Activity of therapeutic JAK 1/2 blockade in graft-versus-host disease. Blood.

[13] Zeiser R, Burchert A, Lengerke C, Verbeek M, Maas-Bauer K, Metzelder SK, et al. Ruxolitinib in corticosteroid-refractory graft-versus-host disease after allogeneic stem cell transplantation: a multicenter survey. Leukemia. 2015;29:2062–8. 10.1038/leu.2015.212 PMC4854652 26228813

[14] Zeiser Robert, Burchert Andreas, Lengerke Claudia, Verbeek Mareike, Maas-Bauer Kristina, Metzelder Stephan, Spoerl Silvia, Ditschkowski Markus, Ecsedi Matyas, Sockel Katja, Ayuk Francis, Salem Ajib, Sicre de Fontbrune Flore, Na Il-Kang, Livius Penter, Holtick Udo, Wolf Dominik, Schuler Esther, Meyer Everett, Apostolova Petya, Bertz Hartmut, Marks Reinhard, Lübbert Michael, Wäsch Ralph, Scheid Christof, Stölzel Friedrich, Ordemann Rainer, Bug Gesine, Kobbe Guido, Shah Omid, Negrin Robert S., Brune Mats, Spyridonidis Alexandros, Schmitt-Graeff Annette, van der Velden Walter J.F.M., Huls Gerwin, Mielke Stephan, Grigoleit Goetz Ulrich, Kuball Jurgen, Flynn Ryan P, Ihorst Gabriele, Du Jing, Blazar Bruce R., Arnold Renate, Kröger Nicolaus, Passweg Jakob R., Halter Joerg, Socié Gérard, Beelen Dietrich W., Peschel Christian, Neubauer Andreas, Finke Jürgen, Duyster Justus, von Bubnoff Nikolas (2016). Long-Term Follow-up of Patients with Corticosteroid-Refractory Graft-Versus-Host Disease Treated with Ruxolitinib. Blood.

[15] Khoury HJ, Langston AA, Kota VK, Wilkinson JA, Pusic I, Jillella A (2018). Ruxolitinib: a steroid sparing agent in chronic graft-versus-host disease. Bone Marrow Transplant.

[16] Przepiorka D, Weisdorf D, Martin P, Klingemann HG, Beatty P, Hows J (1995). 1994 Consensus Conference on Acute GVHD Grading. Bone Marrow Transplant.

[17] Greinix HildegardT (2011). Diagnosis and staging of chronic graft-versus-host disease in the clinical practice. Biol Blood Marrow Transplant.

[18] Lee Stephanie J., Wolff Daniel, Kitko Carrie, Koreth John, Inamoto Yoshihiro, Jagasia Madan, Pidala Joseph, Olivieri Attilio, Martin Paul J., Przepiorka Donna, Pusic Iskra, Dignan Fiona, Mitchell Sandra A., Lawitschka Anita, Jacobsohn David, Hall Anne M., Flowers Mary E.D., Schultz Kirk R., Vogelsang Georgia, Pavletic Steven (2015). Measuring Therapeutic Response in Chronic Graft-versus-Host Disease. National Institutes of Health Consensus Development Project on Criteria for Clinical Trials in Chronic Graft-versus-Host Disease: IV. The 2014 Response Criteria Working Group Report. Biology of Blood and Marrow Transplantation.

[19] Shulman HM, Kleiner D, Lee SJ, Morton T, Pavletic SZ, Farmer E (2006). Histopathologic diagnosis of chronic graft-versus-host-disease: National Institutes of HealthConsensus Development Project on Criteria for Clinical Trials in Chronic Graft-versus-Host Disease: II. Pathology Working Group Report. Biol Blood Marrow Transplant.

[20] Hurabielle C, Sicre de Fontbrune F, Moins‐Teisserenc, Robin M, Jachiet M,Coman (2017). Efficacy and tolerance of ruxolitinib in refractory sclerodermatous chronic graft‐versus‐host disease. Br J Dermatol.

[21] Benden C (2017). Haughton m, Leonard S, Huber LC. Therapy options for chronic lung allograft dysfunction–bronchiolitis obliterans syndrome following first-line immunosuppressive strategies: a systematic review. J Heart Lung Transplant.

[22] Koreth J, Matsuoka K, Kim HT, McDonough SM, Bindra B, Alyea EP (2011). Interleukin-2 and regulatory T cells in graft-versus-host disease. N Engl J Med.

[23] Cutler C, Miklos D, Kim HT, Treister N, Woo SB, Bienfang D (2006). Rituximab for steroid-refractory chronic graft-versus-host disease. Blood.

[24] Benito AI, Furlong T, Martin PJ, Anasetti C, Appelbaum FR, Doney K (2001). Sirolimus (rapamycin) for the treatment of steroid-refractory acute graft-versus-host disease. Transplantation.

[25] Ghez D, Rubio MT, Maillard N, Suarez F, Chandesris MO, Delarue R (2009). Rapamycin for refractory acute graft-versus-host disease. Transplantation.

[26] Hoda D, Pidala J, Salgado-Vila N, Kim J, Perkins J, Bookout R (2010). Sirolimus for treatment of steroid-refractory acute graft-versus-host disease. Bone Marrow Transplant.

[27] Furlong T, Martin P, Flowers ME, Carnevale-Schianca F, Yatscoff R, Chauncey T (2009). Therapy with mycophenolate mofetil for refractory acute and chronic GVHD. Bone Marrow Transplant.

[28] Bacigalupo A, Deeg J, Caballero D, Gualandi F, Raiola AN, Varaldo R (2016). Treatment of patients with steroid refractory acute graft vs host disease (SR-GvHD): a matched paired analysis of anti-CD26 (begelomab) compared to Other Treatment. Blood.

[29] Chen Xiaomei, Wang Chunyan, Yin Jin, Xu Jinhuan, Wei Jia, Zhang Yicheng (2015). Efficacy of Mesenchymal Stem Cell Therapy for Steroid-Refractory Acute Graft-Versus-Host Disease following Allogeneic Hematopoietic Stem Cell Transplantation: A Systematic Review and Meta-Analysis. PLOS ONE.

[30] Miklos D, Cutler CS, Arora M, Waller EK, Jagasia M, Pusic I (2017). Ibrutinib for chronic graft-versus-host disease after failure of prior therapy. Blood.

[31] Greinix HT, Worel N, Just U, Knobler R (2014). Extracorporeal photopheresis in acute and chronic graft-versus-host disease. Transfus Apher Sci.

[32] Hannani D (2015). Extracorporeal photopheresis: tolerogenic or immunogenic cell death? beyond current dogma. Front Immunol.

[33] Heine A, Held SA, Daecke SN, Wallner S, Yajnanarayana SP, Kurts C (2013). The JAK-inhibitor ruxolitinib impairs dendritic cell function in vitro and in vivo. Blood.

